# Recurrent transplant renal vein thrombosis caused by May-Thurner syndrome: a case report and review of literature

**DOI:** 10.1016/j.eucr.2026.103516

**Published:** 2026-06-23

**Authors:** Koosha kamali, Hossein Saffari, Ehsan Zolfi, Shervin Najafizadeh, Nasrollah Abian, Farhad Ahmadi, Vahid Vahedisoraki

**Affiliations:** a Department of Urology, Hasheminejad Kidney Center, School of Medicine, Iran University of Medical Sciences, Tehran, Iran; b Department of Vascular Surgery, Hasheminejad Kidney Center, School of Medicine, Iran University of Medical Sciences, Tehran, Iran

## Abstract

Transplant renal vein thrombosis (TRVT) affects 0.1% to 4.2% of transplants, leading to graft loss in almost all cases. Mechanical factors are the most common underlying cause of TRVT, a category that includes May-Thurner syndrome (MTS). We report a 62-year-old man underwent left-sided kidney transplantation but developed recurrent deep vein thrombosis and TRVT. Two emergency thrombectomies restored graft function, though no iatrogenic cause was identified. Further investigations revealed MTS as the underlying etiology. Left common iliac vein stenting with long-term anticoagulation was implemented, highlighting the importance of considering anatomic venous anomalies in transplant recipients with unexplained or recurrent thrombosis.

## Highlights

• MTS may lead to transplant renal vein thrombosis (TRVT).
• Recurrent TRVT despite anticoagulation can be a sign of MTS.
• Balloon dilation and venous stenting ensuing thrombectomy restores graft function.
• Early recognition and correction of MTS can prevent graft loss.

## 1 Introduction

May–Thurner syndrome (MTS) is characterized by compression of the iliocaval venous segment against the underlying vertebral body by the overlying arterial system, most commonly involving the left common iliac vein (LCIV). Although asymptomatic in most individuals, MTS can impair venous outflow and predispose patients to deep vein thrombosis (DVT).^1^

Transplant renal vein thrombosis (TRVT), which affects 0.1%–4.2% of kidney transplant recipients, occurs predominantly within the first 2 weeks after transplantation surgery^2^^,^^3^ and results in graft loss in most cases.^2^ Mechanical factors, including external compression of the venous system, are considered the most common contributors to TRVT.^2^^,^^4^ Therefore, May-Thurner syndrome (MTS) may predispose patients undergoing left-sided kidney transplantation to TRVT. However, reports of TRVT secondary to MTS in the English-language literature remain scarce. Here, we report a rare case of recurrent TRVT due to MTS and discuss its clinical presentation, diagnostic challenges, and management. We also provide a literature review in this regard.

## 2 Case presentation

A 62-year-old man with autosomal dominant polycystic kidney disease who was on hemodialysis for 2 years and had left nephrectomy one year prior due to severe flank pain, underwent living donor kidney transplantation on the left side of the pelvis. The transplant renal vein (TRV) and artery were anastomosed end-to-side to the left external iliac vein (LEIV) and artery, respectively. Immediate postoperative Doppler ultrasonography (DUS) showed good blood flow. Urine output was 700 mL/hour on the surgery day. Immunosuppressive medications included tacrolimus and prednisolone. By postoperative day (POD) 4, his serum creatinine decreased to 1.3 mg/dL.

On the morning of POD 5, the patient developed left leg swelling. DUS revealed left lower extremity DVT extending to the femoral vein sparing LEIV and TRV. Heparin infusion (1100 units/hour) was started. As the patient complained of surgical site bulging and pain, abdominopelvic CT scan was taken which showed no significant hematoma nor lymphocele. Urine output remained ∼250 mL/h but later that night he became anuric. Repeat DUS showed thrombus progression to the LEIV, TRVT, and severe reduction in transplant renal artery blood flow. Creatinine rose to 3.2 mg/dL.

Urgent surgical exploration revealed a congested graft with palpable thrombosis in the TRV and LEIV. Incising the LEIV proximal to the anastomosis, thrombi burst out. The graft color normalized and no anastomotic stenosis was detected. The incision was sutured and intraoperative DUS confirmed restored blood flow. Heparin was continued postoperatively. The patient's urine output improved, leg swelling resolved, and creatinine returned to 1.4 mg/dL after 24 hours.

2 days later, apixaban 5 mg twice daily replaced heparin. The following day was uneventful. However, on the next day, the patient again developed leg swelling, anuria, and increased creatinine (2.3 mg/dL). DUS confirmed recurrent leg thrombosis extending to LEIV and TRV. A second open thrombectomy was performed. Both heparin and apixaban were continued afterward. Creatinine normalized after 3 days, and follow-up DUS showed no thrombosis. The patient remained hospitalized for further investigations.

Reviewing our case in a multidisciplinary meeting with the presence of transplant surgery team, nephrologist, vascular surgeon, and radiologist, the compression of the LCIV by the left common iliac artery could be inferred from the CT scan (Fig. 1a and b) which suggested the possibility of MTS. Hence, CT venography by injecting contrast into the left femoral vein was proposed for the patient showing contrast in the collateral thigh and pelvic veins evading LEIV, corroborating high pressure in left iliac veins (Fig. 1c); further confirming presence of MTS. Therefore, balloon dilatation and stenting of the LCIV were planned on the same admission session.

**Fig. 1:**
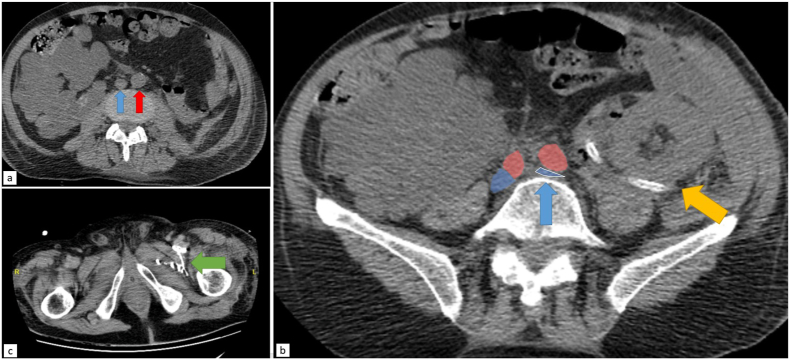
**a.** Abdominopelvic CT scan without contrast showing inferior vena cava (blue arrow) and aorta (red arrow). **b.** Abdominopelvic CT scan without contrast indicating right and left common iliac arteries (marked by red color), right common iliac vein (marked by blue color), and left common iliac vein (marked by blue color and white outline). The compression of left common iliac vein by left common iliac artery is evident (blue arrow). The transplanted kidney and the drain can be seen in the image (yellow arrow). **c.** Pelvic CT venography revealing contrast entrance into small veins as collateral veins after injection of contrast into left femoral vein (green arrow).

During the intervention, the left femoral vein access was obtained. Venography confirmed LCIV stenosis and dilated collateral veins (Fig. 2a). Balloon angioplasty was performed by balloon dilatation catheter (Atlas™16mm-40mm). Then, iliac vein stent (Venovo™18mm-120mm) was deployed from iliocaval confluence to LEIV proximal to the anastomosis. Post-deployment dilatation was completed and subsequent venography showed normal contrast flow into the LCIV and inferior vena cava, with near resolution of collateral veins (Fig. 2b and c).

**Fig. 2:**
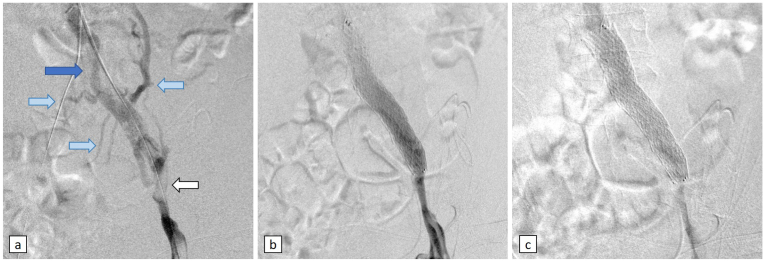
**a.** Venography revealed left common iliac vein stenosis by relatively less contrast entering it (dark blue arrow) and more contrast entering collateral veins (light blue arrows) after injection from left femoral vein. White arrow depicts the location of graft vein anastomosis. **b.** Contrast passage from left femoral vein to left external iliac vein and left common iliac vein after deployment of venous stent. **c.** Contrast was washed away from left external iliac vein and left common iliac vein into inferior vena cava without entering collateral veins after deployment of venous stent.

The patient was discharged 6 days after venous stenting on apixaban having creatinine of 1.4 mg/dL. 4 months later, he remained symptom-free with normal graft function (creatinine 1.3 mg/dL) and no recurrence of DVT or TRVT.

## 3 Discussion

MTS was first described in 1957 as a cause of pelvic vein thrombosis.^5^ A decade later, Cockett and Thomas established its connection to ipsilateral iliofemoral DVT.^6^ The exact prevalence of MTS in general population remains unclear as MTS is often asymptomatic.^7^ It is clinically linked to 2 to 5% of all DVT cases; however, MTS may have a more significant role given the disproportionately higher incidence of left-sided DVT.^8^

TRVT presents acutely with symptoms such as oliguria, deteriorating graft function, and painful graft, which in severe cases may lead to its rupture.^3^ Among numerous risk factors for TRVT, mechanical causes, including venous compression, are considered significant contributors^2^^,^^3^^,^^7^; and as MTS is a form of venous compression, it should be recognized as an important risk factor for TRVT. In Table 1 we categorized all possible risk factors for TRVT.^2^^,^^3^^,^^7^

**Table 1:** Risk factors of transplant renal vein thrombosis.

Category	Risk Factors	Mechanisms/Notes
**Donor-related**	Use of right kidney	Shorter renal vein (prone to compression)
	Multiple graft vessels	Associated in some studies, disputed in others
	Prolonged ischemic time	Endothelial thrombogenicity, Decreased perfusion due to acute tubular necrosis may cause thrombosis
	Vascular injuries	Endothelial damage increases thrombosis risk
	Older donor age	Atherosclerotic vessels
**Recipient-related**	Atherosclerosis (esp. in older recipients)	Atherosclerotic vessels predisposes to thrombosis
	Vessel size mismatch (esp. in pediatric patients)	Small recipient vessels predispose to thrombosis
	Pre-transplant dialysis modality	Peritoneal dialysis increases plasma procoagulant factors
	Hypercoagulable states	Antiphospholipid antibody syndrome, antithrombin deficiency, Factor V Leiden mutation, prothrombin gene mutation
	Perioperative hemodynamic instability	Hypotension or dehydration may induce hypoperfusion of kidney and thrombosis
	Primary renal disease	Membranous nephropathy links with higher risk of venous graft thrombosis
	Inadequate recipient's venous flow	Stenosis of iliac vein such as May-Thurner syndrome
	Extension of pre-existing deep vein thrombosis	May propagate into graft vein
**Technical (operative)**	Kinking of graft vein	
	Long renal vein	More prone to compression
	Anastomotic stenosis	Narrowing predisposes to clot formation
	Endothelial injury during surgical manipulation	
	Compression	Hematomas, lymphoceles, or renal artery compression (esp. contralateral transplants)
**Immunosuppression-related**	Calcineurin inhibitors (general)	Promote hypofibrinolysis via enhancing plasminogen activator inhibitor expression
	CD3 Monoclonal Antibody	
	High-dose methylprednisolone pulses	
	Antithymocyte/antilymphocyte globulin	

Ultrasonography is the first-line imaging modality for diagnosing TRVT. Gray-scale ultrasonography may show cortical edema, perirenal hematoma, and TRV thrombosis. DUS can detect venous obstruction signs namely reversed arterial diastolic flow, a spike-like systolic wave, and absence of renal vein visualization.^2^ Advanced imaging such as CT angiography (CTA) offers superior anatomical detail and can identify rare anomalies like MTS. Although conventional angiography remains the gold standard for vascular diagnosis, its invasiveness and risk of nephrotoxicity restrict its use to patients planned for endovascular interventions.^2^^,^^3^

We summarized cases of MTS affecting transplanted kidney in Table 2. Among these cases, four were successfully managed with endovascular balloon dilation and stenting. One case, however, underwent graftectomy due to severe graft damage. What makes our case special is the recurrence of DVT and TRVT despite anticoagulation therapy, necessitating thrombectomy twice. Such phenomenon combined with the fact that proximal extension of an iliofemoral DVT is not expected due to the high volume of blood entering the iliac vein from the graft,^9^ guided us to look for an underlying pathology found to be MTS which was, subsequently, treated with balloon dilatation and LCIV and LEIV stenting.

**Table 2:** Review of all cases of May-Thurner syndrome affecting transplanted kidney in English literature.

Author (year)	Age, Sex, Past medical history	Living vs Deceased donor kidney	Side	Iliac vessels used for anastomosis	Anticoagulant after surgery	Immunosuppression	Interval from surgery to disease presentation	Final diagnosis	TRVT	Left lower extremity edema or DVT	Treatment	Final Cr level/other outcome
Arrazola et al. (2001)^7^	63 years, Male, DM, HTN, CAD, PVD	Living	Left	LEIA and LEIV	low-dose heparin drip (200 units/hour)	antibody induction plus MMF, tacrolimus, and prednisone	2 days	2 different areas of iliac vein compression: 1) LEIV by hematoma 2) LCIV due to MTS	No	No	Deploying 2 stents: one in LEIV and the other one in LCIV	1.9 mg/dL on discharge day (10 days after procedure)
Campsen et al. (2010)^11^	57 years, Female	Living	Left	Not specified	-	N/A	13 days	Fixed stenosis at the origin of the LCIV	Yes	Yes	Thrombectomy with balloon maceration, deploying iliac vein stent	1.3 mg/dL on discharge day (3 days after procedure)
Vaidya et al. (2012)^12^	68 years, Female, ADPKD	Deceased	Left	Not specified	-	tacrolimus and prednisone	4 months	LCIV compression by right iliac artery	Yes	No	Thrombolysis, Angioplasty and deploying iliac vein stent	0.8 mg/dL after 1 year
Konopa et al. (2024)^3^	63 years, Male	Deceased	Left	LEIA and LEIV	Low molecular weight heparin	MMF, tacrolimus, and prednisone	4 days	Narrowed LCIV due to MTS	Yes	No	Graftectomy	Second kidney transplant surgery on right side 18 months later
Onuigbo et al. (2025)^13^	43 years, Male, ADPKD	Deceased	Left	Not specified	-	N/A	2 months	Narrowed LEIV compressed by native ADPKD kidney	No	No	open left native nephrectomy	2.0 mg/dL
Kamali et al. (2025)-current case	62 years, Male, ADPKD	Living	Left	LEIA and LEIV	-	tacrolimus and prednisone	5 days	LCIV compression by right iliac artery	Yes	Yes	Open thrombectomy (twice), LCIV balloon dilation with deploying stent into LCIV and LEIV	1.3 mg/dL four months after surgery

**Abbreviations:** ADPKD, autosomal dominant polycystic kidney disease; CAD, coronary artery disease; Cr, creatinine; DVT, deep vein thrombosis; DM, diabetes mellitus; DUS, Doppler ultrasonography; HTN, hypertension; LCIV, left common iliac vein; LEIA, left external iliac artery; LEIV, left external iliac vein; MRA, magnetic resonance angiography; MTS, May-Thurner syndrome; MDM, multidisciplinary meeting; MMF, Mycophenolate mofetil; N/A, not available; PVD, peripheral vascular disease; POD, post-operation day; TRV, transplant renal vein; TRVT, transplant renal vein thrombosis; U/O, urine output.

As Table 2 depicts, endovascular treatment including balloon dilatation and stenting (with thrombectomy or thrombolysis whenever needed) seems to be a promising approach for managing MTS-related TRVT. Our unique experience in this patient confirms that urgent thrombectomies can save transplanted kidney before stent placement, but it is the balloon dilation and stenting which ultimately prevents recurrent thrombosis.

There is currently no literature supporting prophylactic stent placement in transplant candidates with MTS.^10^ However, given the potential “one too many” complication, i.e. graft loss, prophylactic stenting may be a reasonable strategy. Future studies may provide insight regarding the prophylactic role of such intervention in MTS patients undergoing left side kidney transplantation.

## 4 Conclusion

TRVT, especially recurrent one, may be secondary to unrecognized venous anomalies such as MTS. In such cases, early diagnosis and correction with thrombectomy plus iliac vein stenting can preserve graft function and prevent TRVT recurrence.

## CRediT authorship contribution statement

**Koosha kamali:** Supervision, Methodology, Conceptualization. **Hossein Saffari:** Validation, Data curation. **Ehsan Zolfi:** Visualization, Investigation. **Shervin Najafizadeh:** Writing – review & editing, Supervision. **Nasrollah Abian:** Writing – original draft, Validation, Software, Investigation. **Farhad Ahmadi:** Investigation, Data curation. **Vahid Vahedisoraki:** Writing – review & editing, Investigation.

## Funding sources

This research did not receive any specific grant from funding agencies in the public, commercial, or not-for-profit sectors.

## Declaration of competing interest

The authors declare that they have no known competing financial interests or personal relationships that could have appeared to influence the work reported in this paper.
